# Cigarette smoke exposure impairs early‐stage recovery from lengthening contraction‐induced muscle injury in male mice

**DOI:** 10.14814/phy2.70064

**Published:** 2024-09-27

**Authors:** Nicole E. Stevens, Mafalda Loreti, Israel Ramirez‐Sanchez, Felipe C. G. Dos Reis, Alessandra Sacco, Ellen C. Breen, Leonardo Nogueira

**Affiliations:** ^1^ Section of Physiology, Division of Pulmonary, Critical Care and Sleep Medicine, Department of Medicine University of California San Diego La Jolla California USA; ^2^ Development, Aging and Regeneration Program Sanford Burnham Prebys Medical Discovery Institute La Jolla California USA; ^3^ Division of Cardiovascular Medicine, Department of Medicine University of California San Diego La Jolla California USA; ^4^ Seccion de Estudios de Posgrado e Investigacion Escuela Superior de Medicina, IPN Mexico City Mexico; ^5^ Division of Endocrinology and Metabolism, Department of Medicine University of California San Diego La Jolla California USA; ^6^ School of Exercise and Nutritional Sciences, College of Health and Human Services San Diego State University San Diego California USA

**Keywords:** eccentric contractions, myofiber growth, sustained inflammation, tobacco smoke

## Abstract

The use of tobacco cigarettes produces locomotor muscle weakness and fatigue intolerance. Also, smokers and chronic obstructive pulmonary disease patients have a greater incidence of muscle injury and a deficient myogenic response. However, the effects of smoke exposure on the recovery from eccentric exercise‐induced muscle injuries are unknown. Mice were exposed daily to cigarette smoke (CS) or room air (Air) for 4 months; the anterior crural muscles from one limb were injured by a lengthening contractions protocol (LCP) and recovered for 7 days. Lung compliance was greater, and body weights were lower, in CS‐exposed than in the Air group. In LCP‐subjected limbs, CS exposure lowered *tibialis anterior* myofiber cross‐sectional area, decreased the size of centrally nucleated myofibers, and decreased *extensor digitorum longus* (EDL) mass, but did not affect EDL force from both limbs. CS exposure upregulated the mRNA levels of several myogenic (*Pax7, Myf5, nNOS*) genes in the EDL. The combination of CS exposure and LCP decreased *Myf5* and *nNOS* mRNA levels and exacerbated pro‐inflammatory mRNA levels. These data suggest that smoke exposure leads to an excessive pro‐inflammatory response in regenerating muscle that is associated with a lower muscle mass recovery from a type of injury that often occurs during strenuous exercise.

## 1 INTRODUCTION

It is widely accepted that long‐term smoking is the leading preventable cause of chronic obstructive pulmonary disease (COPD) (Leberl et al., 2013), a disease characterized by irreversible pulmonary airflow limitation and abnormal inflammation of the lungs (Pauwels et al., 2001). Smokers and COPD patients also experience adverse extrapulmonary effects that are detected before the onset of overt respiratory limitations (Degens et al., 2015; Morse et al., 2007). Studies of chronic smokers and rodents exposed for several months to cigarette smoke (CS) reveal locomotor muscles with reduced force‐generating capacity due to muscle atrophy (Chan et al., 2020; Gosker et al., 2009; Peinado et al., 2023) and decreased fatigue resistance. The latter is thought to develop in part due to a loss of fatigue‐resistant myofibers (Chan et al., 2020; Tang et al., 2010), a slowing in myofiber intracellular Ca^2+^‐handling (Nogueira et al., 2018), and skeletal muscle capillary regression (Nogueira et al., 2018; Tang et al., 2010). All of these aberrant skeletal muscle changes could contribute to exercise intolerance in smokers and COPD patients (Degens et al., 2015).

Another limitation of smokers and COPD patients, which has been less well investigated, is a higher incidence of muscle injury (Baumgarten et al., 2010; Bishop et al., 2015; Brooks et al., 2019; Orozco‐Levi et al., 2012). Muscle satellite cell (MuSC) populations, which are important for the maintenance and repair of skeletal muscle fibers, have a reduced proliferative capacity in smokers (Pomies et al., 2015; Theriault et al., 2012, 2014). Whether changes in MuSC fate impede muscle regenerative capacity and prolong the repair time after injury in smokers and COPD patients has not been thoroughly investigated. Data from our group suggest that mice exposed to periods of CS over several months decrease the pool of quiescent MuSC (Nogueira et al., 2018). The effect of CS exposure on muscle regeneration has been tested in mice chemically injured by continuous depolarization of myofibers with barium chloride (BaCl_2_) (Chan et al., 2020). In this study, as reported by Chan et al. (Chan et al., 2020), muscle force and MuSC activation were impaired within the first 7 days of recovery following intramuscular BaCl_2_ injections. These data suggest a negative impact of CS exposure on early‐stage muscle regeneration.

Non‐physiologic injuries produced by myotoxins (e.g., BaCl_2_ or cardiotoxin) have been widely used to study the function of MuSC during muscle regeneration since they produce large spontaneous myofiber degeneration followed by a well‐defined process of regeneration (Morton et al., 2019; Tierney & Sacco, 2016). However, the most common types of muscle injuries that occur in humans are exercise‐induced muscle strains from strenuous eccentric exercise. These injuries account for about 30% of all sports‐related injuries (Ekstrand et al., 2011; Garrett Jr., 1996; Jarvinen et al., 2005). Furthermore, smokers and COPD patients are more susceptible to this type of overuse injury (Baumgarten et al., 2010; Brooks et al., 2019). However, the extent to which chronic smoke exposure affects skeletal muscle function following an exercise‐induced strain remains unknown.

Overuse muscle injury can be modeled in experimental animals by subjecting muscles to lengthening contractions during electrical nerve stimulation. We hypothesized that chronic exposure to CS delays eccentric exercise‐induced recovery of locomotor muscle force and myofiber size by inhibiting the early‐stage myogenic regenerative response. To test this hypothesis, wild‐type mice were exposed to daily periods of smoke from reference tobacco cigarettes or room air (Air) over 4 months. At the end of the 4‐month exposure period, the anterior crural muscles (ACM) from one leg were subjected to lengthening contractions. Seven days later, the *extensor digitorum longus* (EDL) and *tibialis anterior* (TA) muscles were evaluated for early‐stage changes in ex‐vivo muscle function, myofiber cross‐sectional area, newly formed myofibers, and myogenic gene expression. The muscles in the contralateral leg served as non‐injured controls.

## 2 MATERIALS AND METHODS

### 2.1 Ethical approval

All procedures were approved by the University of California, San Diego Institutional Animal Care and Use Committee (UCSD‐IACUC, Protocol # S00250) and San Diego State University IACUC (Protocol # 22‐04‐003N) and comply with the American Physiological Society's Guiding Principles in the Care and Use of Vertebrate Animals in Research and Training. Wild‐type (C57BL/6J) mice, ages 7–8 weeks (total of 24 mice), from Jackson Laboratory (Bar Harbor, ME; cat# 000664) were used in the present study. Only male mice were used. Mice were kept on a 12‐h:12‐h light/dark cycle, and regular chow (Teklad Global 14% Protein Rodent Maintenance Diet, Cat# 2014, Inotiv, West Lafayette, IN) and water were provided ad libitum. When mice were subjected to the lengthening contractions protocol to produce muscle injury, animals were anesthetized with isoflurane (1.5%–2.5% v/v). Mice were anesthetized with ketamine and xylazine (10:1 mg/kg, i.p.) for in vivo lung mechanical functional analysis and blood and tissue collection. After tissue collection, euthanasia was ensured by surgical removal of the heart.

### 2.2 Cigarette smoke (CS) or air exposure

Mice were randomly assigned to Air (*n* = 12 mice) or CS (*n* = 12 mice) exposure groups. Mice were exposed using a “nose‐only” system (InExpose, SCIREQ, Montreal, QC, Canada) over 16 weeks with 5 days of pre‐acclimatization in the restraints as previously described (Nogueira et al., 2018). Smoke was obtained from burning un‐filtered 1R6F research cigarettes (Kentucky Tobacco Research and Development Center, University of Kentucky, Lexington, KY) by a negative pressure pump set at 3 L.min^−1^ with the motor programmed to pull CS for 2 s with 58 s of fresh air, to simulate a smoke “puff” and ensuring safe delivery of smoke to mice. Mice were exposed 5 days each week to CS for two consecutive sessions of five cigarettes each (~50 min each session, 10 cigarettes total) with a 30‐min period of fresh air between sessions (Nogueira et al., 2018). Smoke exposure was assessed by measuring the amount of cotinine in the plasma 24 h after the last exposure by ELISA (Cat# EA100902, OriGene, Rockville, MD).

### 2.3 Lengthening contraction protocol (LCP)

One week before the end of the exposure period (Week 15 of the exposure), the ACM from the right hindlimb were subjected to an LCP to injure the TA and EDL muscles (Ingalls et al., 1998; Lovering et al., 2009). The contralateral leg (left hindlimb) served as a non‐injured control. Mice were placed on a water‐heated surgical platform at 37–38°C and kept anesthetized with oxygenized isoflurane vapor (~2% v/v) during the experimental procedure. The right lower leg was shaved, and the knee was securely fixed by inserting a sterile 27‐gauge, 1.5‐inch needle under the patella. The foot was attached to a lever plate connected to a torque transducer (Model# QWFK‐8 M, Honeywell Inc., Golden Valley, MN) and to a digital high‐speed servo motor (Model# HS‐5625MG, Hitec RCD Inc., Poway, CA), and the ankle was positioned at a 90° angle. Disposable ultra‐subdermal needle electrodes (Natus Medical Inc., Middleton, WI) were subcutaneously placed near the head of the tibia to electrically stimulate the peroneal nerve using a S48 stimulator (Grass Technologies, Quincy, MA) to evoke contractions of the ACM. Optimal voltage (2–6 V) was determined by measuring the maximal peak torque obtained while evoking tetanic contractions (100 Hz pulse‐frequency, 600 ms trains, 0.1 ms pulses) once each minute. For the LCP, an Arduino Uno board (Arduino S.R.L.) controlled the activation and range of motion of the servomotor, the activation of the electrical stimulator at each contraction, and the intervals between contractions. The board was programmed to activate the movement of the servo motor 200 ms after the beginning of each contraction. Stimulator parameters such as train duration, pulse duration, and voltage were set directly on the electrical stimulator. LCP was initiated by electrically stimulating the peroneal nerve at 150 Hz pulse‐frequency (600 ms trains, 0.1 ms pulses), which produced a near‐maximal torque production of the ACM, followed by an active 90° plantar flexion after 200 ms of the beginning of the 600‐ms train at 250°/s. Contractions were repeated every 12 s for 30 min (a total of 150 lengthening contractions). Torque signals (in N.mm) were normalized by the mouse body weight and reported as Nmm/kg. After the LCP, mice recovered for 7 days before in vivo lung compliance and tissue collection measurements. Mice continued to be exposed to Air or CS during the 7‐day recovery period.

### 2.4 Lung mechanical properties

Static lung mechanics were evaluated by measuring the pressure‐volume relationship using a FlexiVent apparatus (SCIREQ, Montreal, QC, Canada), as previously reported (Lee et al., 2019). Briefly, mice were anesthetized (i.p.) with ketamine and xylazine, tracheostomized, and an 18G cannula (0.5 inches length) positioned in the trachea. Airway pressure was recorded as the lungs were inflated and deflated at 0.1 mL intervals with a maximum pressure of 30 cmH_2_O. Total lung capacity [TLC], residual volume [RV], and lung compliance [C] were determined according to Limjunyawong et al. (Limjunyawong et al., 2015).

### 2.5 Skeletal muscle ex‐vivo contractility of the EDL

EDL muscles from both hindlimbs were dissected and mounted horizontally in a muscle strip myograph system (800MS; Danish Myo Technology, Aahus, Denmark). The EDL was perfused with Tyrode solution (in mM: 121 NaCl, 5 KCl, 0.4 NaH_2_PO_4_, 1.8 CaCl_2_, 0.5 MgCl_2_, 24 NaHCO_3_, 5.5 glucose, 0.1 EGTA, 25 μM (+)‐Tubocurarine hydrochloride) that was bubbled continuously with 95% O_2_ and 5% CO_2_ (final pH 7.4) at 22°C during the time course of the experiment (Nogueira et al., 2018). Muscle contractions were evoked by electrical stimulation using platinum plates parallel to the muscle length with an S88X stimulator (Grass Technologies, Quincy, MA) at 0.5 ms pulse, 300 ms trains, 16 V. After mounting the muscles, the optimal muscle length (L_0_) was determined by single twitches and rested for 10 min. Contractions were evoked at different frequencies of pulse‐stimulation (1–150 Hz, 1 contraction each 100 s) to determine the excitation–contraction coupling properties and maximal force produced by EDL muscles from LCP‐subjected legs (LCP) and contralateral legs (control) and force was measured at 500 Hz acquisitions. To determine muscle force recovery after the 7‐day recovery period, absolute force (in mN) produced by the EDL from the LCP leg was normalized by the maximal force produced by the control muscle from the same mouse. EDL muscles were frozen in liquid N_2_ and kept at −80°C for later gene expression analysis.

### 2.6 Morphometric analysis of TA cross‐sections

TA muscles were embedded in optimal cutting temperature compound (O.C.T.), frozen in liquid‐N_2_‐cold isopentane, and stored at −80°C. TA muscle sections of 10 μm thickness were mounted on Fisherbrand™ Superfrost™ Plus Microscope Slides (Thermo Fisher Scientific Cat# 22‐037‐246) and fixed with 4% paraformaldehyde (PFA) at room temperature (RT) for 15 min. Slides were blocked with AffiniPure Fab Fragment Goat Anti‐Mouse IgG (H + L) antibody (diluted 1:10, Jackson ImmunoResearch Labs Cat# 115‐007‐003, RRID: AB_2338476) in PBST‐B (1× PBS, 0.1% v/v Triton‐100, 2% w/v Bovine Serum Albumin fraction V) for 30 min at RT. After blocking, a group of slides was incubated for 1 h at RT with primary antibody for rat anti‐laminin B2 monoclonal antibody (diluted 1:100, Millipore Cat# 05–206, RRID: AB_309655) in PBST‐B, followed by 1‐h incubation of secondary antibody Goat anti‐Rat IgG (H + L) Alexa Fluor 546 (diluted 1:250, Thermo Fisher Scientific Cat# A‐11081, RRID: AB_2534125) in PBST‐B. A second group of slides was incubated for 1 h at RT with rat anti‐laminin B2 monoclonal antibody (diluted 1:100) in PBST‐B, 1‐h incubation of goat anti‐rat IgG (H + L) Alexa Fluor 546 (diluted 1:250), fixed again in 4% PFA for 5 min, and antigen retrieval was performed with citrate buffer as described in (Tierney & Sacco, 2016). Slides were incubated with mouse Pax7 monoclonal antibody (Dilution: 1:1, DSHB, Cat# PAX7, RRID: AB_2299243) overnight, followed by 1‐h incubation of secondary antibody goat anti‐mouse IgG1 Alexa Fluor 488 (diluted 1:250, Thermo Fisher Scientific Cat# A‐11001, RRID: AB_2534069) in PBST‐B. Fluorescence was preserved with Prolong Gold antifade reagent with DAPI (Thermo Fisher Scientific Cat# P36935) to detect centrally nucleated myofibers. Images of muscle transverse sections were acquired using an Inverted IX81 Olympus Compound Fluorescence Microscope with ×10 or ×20 magnification lenses, color/monochrome cooled CCD camera, Spot RT3, and MetaMorph 7.11 Software (UIC, Molecular Devices). Measurements of the cross‐sectional area (CSA) of myofibers (1011 ± 263 myofibers per slide, mean ± SD) and quantification of centrally nucleated myofibers were conducted by the same experimenter (N.E.S) using the Fiji (ImageJ) software.

### 2.7 Real‐time PCR from EDL muscle homogenates

After ex‐vivo contractility measurements, EDL muscles (control and LCP) from each group (Air and CS) were randomly used for mRNA measurements. Total RNA was extracted using a RNeasy Plus Universal Mini Kit (Qiagen, Cat# 73404), following the manufacturer's protocol, and RNA yield was measured using a NanoDrop 1000 (Thermo Fisher Scientific, Wilmington, DE). cDNA was synthesized from 500 ng of mRNA using SuperScript III and random hexamers (Invitrogen, Cat# 18080–051). PCR was carried out in 10 μL reactions (2 μL cDNA) using iTaq SYBR Green supermix (Thermo Fisher Scientific, Cat# A46109) on a StepOnePlus Real‐Time PCR Systems (ThermoFisher Scientific). The data presented correspond to the mean of 2^−ΔΔCt^ from at least three independent experiments after being normalized to *GAPDH* (Maurya et al., 2018). The data obtained was then normalized by the mRNA expression of the control muscle of the Air group. qPCR was performed using primers (Integrated DNA Technologies, Coralville, IA), as shown in Table 1.

**TABLE 1:** Primers used for mRNA analysis.

Gene	Accession #	Forward sequence	Reverse sequence
*Atrogin‐1*	AF441120.1	GGAAGGGCACTGACCATCC	GGAAAGTGAGACGGAGCAGC
*GAPDH*	BC023196.2	CCCACTCTTCCACCTTCGATG	GTCCACCACCCTGTTGCTGTAG
*IL‐10*	NM_010548.2	TGAATTCCCTGGGTGAGAAG	TCACTCTTCACCTGCTCCACT
*IL‐1b*	NM_008361.4	GAAATGCCACCTTTTGACAGTG	TGGATGCTCTCATCAGGACAG
*IL‐6*	M20572.1	CCAAGAGGTGAGTGCTTCCC	CTGTTGTTCAGACTCTCTCCCT
*iNOS*	NM_010927.4	TGACCATCATGGACCACCAC	ACCAGCCAAATCCAGTCTGC
*MRF4*	NM_008657.3	CTGCGCGAAAGGAGGAGACTAAAG	ATGGAAGAAAGGCGCTGAAGACTG
*MuRF1*	NM_001039048	CTGCGAATCCCTACTGGACC	CGGAAACGACCTCCAGACA
*Myf5*	NM_008656.5	TGCCATCCGCTACATTGAGAG	CCGGGGTAGCAGGCTGTGAGTTG
*MyoD*	NM_010866.2	GAGCGCATCTCCACAGACAG	AAATCGCATTGGGGTTTGAG
*Myogenin*	NM_031189.2	CCAGTACATTGAGCGCCTAC	ACCGAACTCCAGTGCATTGC
*Myostatin*	NM_010834.3	CAGACCCGTCAAGACTCCTACA	CAGTGCCTGGGCTCATGTCAAG
*nNOS*	NM_008712.3	ACCAGCACCTTTGGCAATGGAG	GAGACGCTGTTGAATCGGACCT
*Pax7*	NM_011039.2	GACTCGGCTTCCTCCATCTC	AGTAGGCTTGTCCCGTTTCC
*TGF‐beta*	NM_011577.2	CTCCCGTGGCTTCTAGTGC	GCCTTAGTTTGGACAGGATCTG

### 2.8 Statistical analyses

All the analyses were conducted using GraphPad Prism® version 10.1.2 for Windows (San Diego, California, USA). For comparisons between multiple groups, two‐way ANOVA or three‐way ANOVA with Bonferroni post hoc tests were used, as indicated in the text and figure legends. For comparisons between two means, unpaired Student's *t*‐test was used as indicated in the text and the figure legends. Statistical significance was accepted when *p* < 0.05. The experimental results in the text and figures are presented as mean ± standard deviation (SD). Investigators were blind during in vivo lung mechanical measurements, tissue collection, ex‐vivo contractile measurements, immunofluorescence analysis, and mRNA analyses.

## 3 RESULTS

### 3.1 Plasma cotinine levels and body weight gain

**FIGURE 1:**
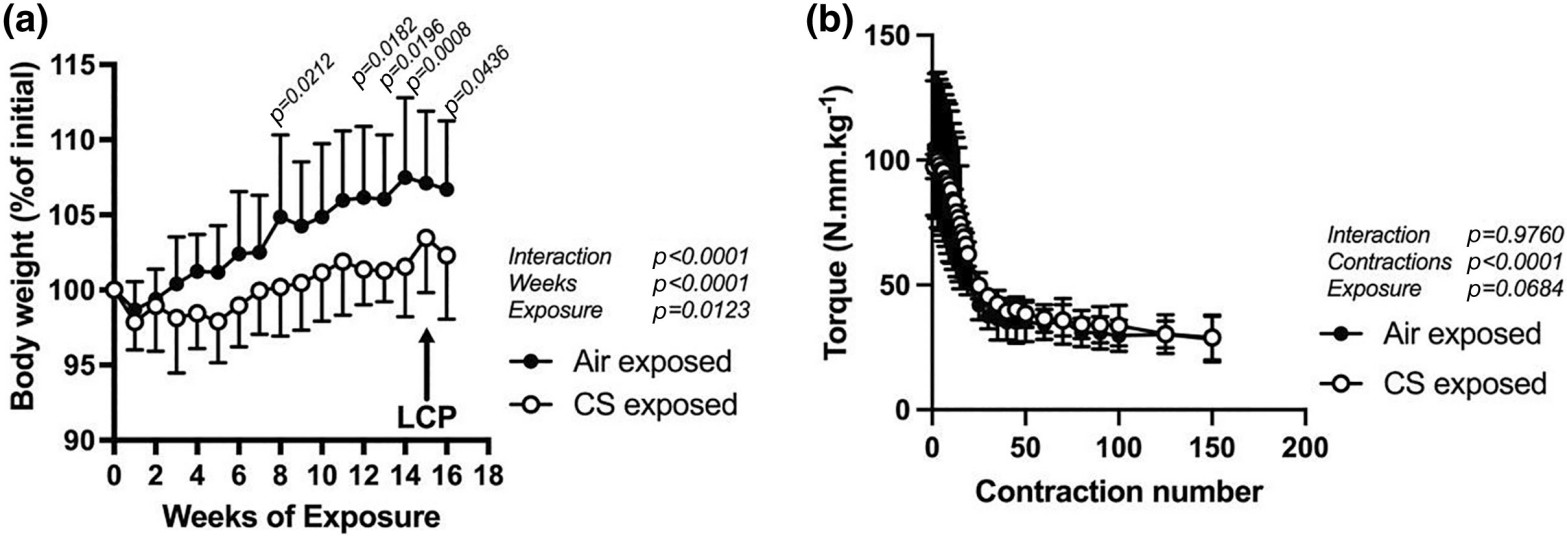
Effects of 4 months of daily CS or Air exposures on body weight and torque responses during lengthening contractions. (a) Weekly body weight gain in Air‐ and CS‐exposed groups during the 16 weeks of exposures. *N* = 12 mice per group (Air or CS), two‐way ANOVA, *p‐*values obtained by the Bonferroni post‐test are shown above time points; (b) Peak torque normalized by mouse body weight (N.mm.kg−1) during LCP performed at Week 15 of exposures (indicated by the arrow in [a]). *N* = 6 mice per group, two‐way ANOVA.

Twenty‐four hours after the last 4‐month exposure to either Air or CS, plasma cotinine levels were higher (*p* < 0.0001, unpaired Student's *t*‐test) in CS‐exposed mice (0.439 ± 0.249 ng/mL, *n* = 10 mice) than Air exposed mice (0.018 ± 0.025 ng/mL, *n* = 11 mice). The mean body weight of the male mice assigned to each exposure group was not different before the exposure period (i.e., 7–8 weeks old, 26.8 ± 2.0 g for Air group vs. 27.7 ± 2.0 g for CS group, *p* = 0.2607, Student *t*‐test, *n* = 12 mice each group). During the 4‐month exposure, Air exposed mice showed a progressive increase in body weight (7 ± 5%, Figure 1a) that was greater than the CS‐exposed mice (2 ± 4%, *p* = 0.012; Two‐way ANOVA), suggesting a suppression in the weight gain in the CS exposed group.

### 3.2 Torque measurements during LCP

To monitor the extent of the initial injury, in vivo decay of isometric ankle torque was recorded during the lengthening contractions. Peak torque before LCP was not different between groups (105 ± 27 vs. 97 ± 5 N.mm/kg, for Air vs. CS groups, respectively, *p* = 0.53; unpaired *t*‐test). Also, the decay in torque during LCP was not different between exposure groups (*p* = 0.31; Two‐way ANOVA, Figure 1b) and, at the 150th contraction, isometric torque was similarly decreased from baseline in the Air and CS exposure groups (Air, 71 ± 11 and CS, 70 ± 10%, *p* = 0.91, unpaired *t*‐test).

### 3.3 Morphometric analysis of myofiber size and myogenic response

**FIGURE 2:**
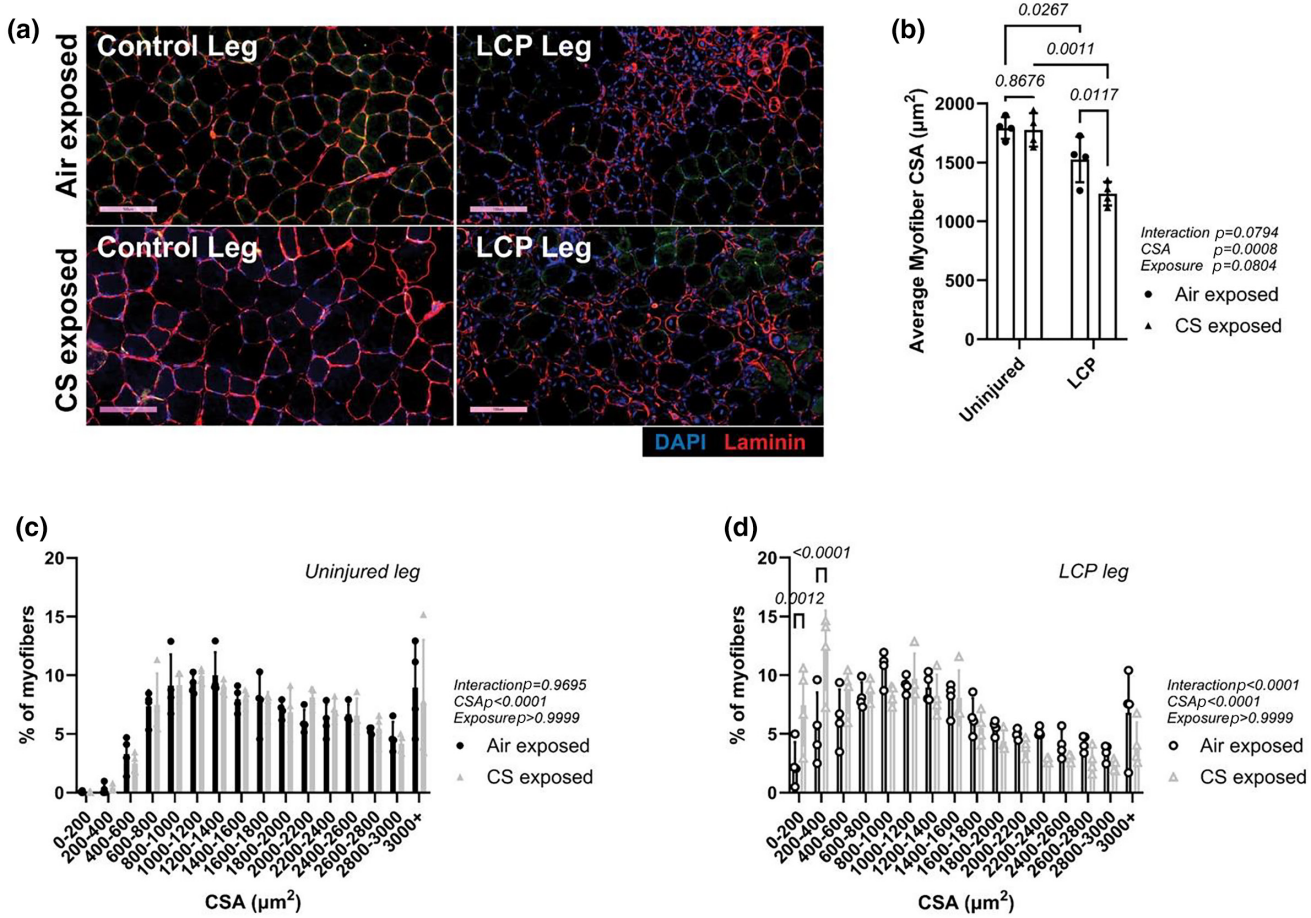
Myofiber cross‐sectional area from *tibialis anterior* (TA) muscles 7 days after lengthening contractions in Air‐ and CS‐exposed mice. (a) Representative immunofluorescence image of TA muscle sections from control and LCP legs from Air and CS exposed groups. Scale bar = 100 μm; (b) Average of myofiber CSA of muscles from control and LCP legs from both exposure groups. *N* = 4 mice per group, two‐way ANOVA repeated measures (control vs. LCP); (c, d) Myofiber CSA distribution in muscles from control legs (c) and LCP legs (d) between Air‐exposed and CS‐exposed groups. *N* = 4 mice per group, two‐way ANOVA. *p*‐values obtained by the Bonferroni post‐test are shown above bars.

Cross‐sectional areas, the number of centrally nucleated myofibers, and the number of Pax7‐positive cells were measured in TA muscles after 7 days of recovery from LCP. As demonstrated in Figure 2a, muscles from LCP‐subjected hindlimbs show areas with centrally nucleated myofibers, indicating tissue regeneration. For all myofibers (i.e., non‐contrally nucleated and centrally nucleated), mean myofiber CSA in the non‐injured TA was not different between exposure groups (Air, 1792 ± 92 μm^2^, CS, 1775 ± 142 μm^2^, *p* = 0.87, Bonferroni post‐test, Figure 2b). Mean myofiber CSA from LCP‐injured TA was smaller than the non‐injured contralateral muscle for both exposure groups (Air, −15 ± 9%, CS −30 ± 9%; *p* = 0.0008 control vs. LCP, two‐way ANOVA, Figure 2b). In addition, myofiber CSA in LCP TA isolated from CS exposed mice were smaller than LCP TA from Air exposed mice (Air, 1525 ± 193 μm^2^, CS, 1235 ± 100 μm^2^; *p* = 0.012, Bonferroni post‐test, Figure 2b). When the myofiber CSA distribution was compared between Air and CS groups in either control (Figure 2c) or LCP legs (Figure 2d), CS exposed mice had a greater fraction of myofibers with smaller CSAs (i.e., 0–400 μm^2^) than Air exposed mice in the LCP‐injured muscle (*p = 0.04, Bonferroni post‐test, Figure 2d).

**FIGURE 3:**
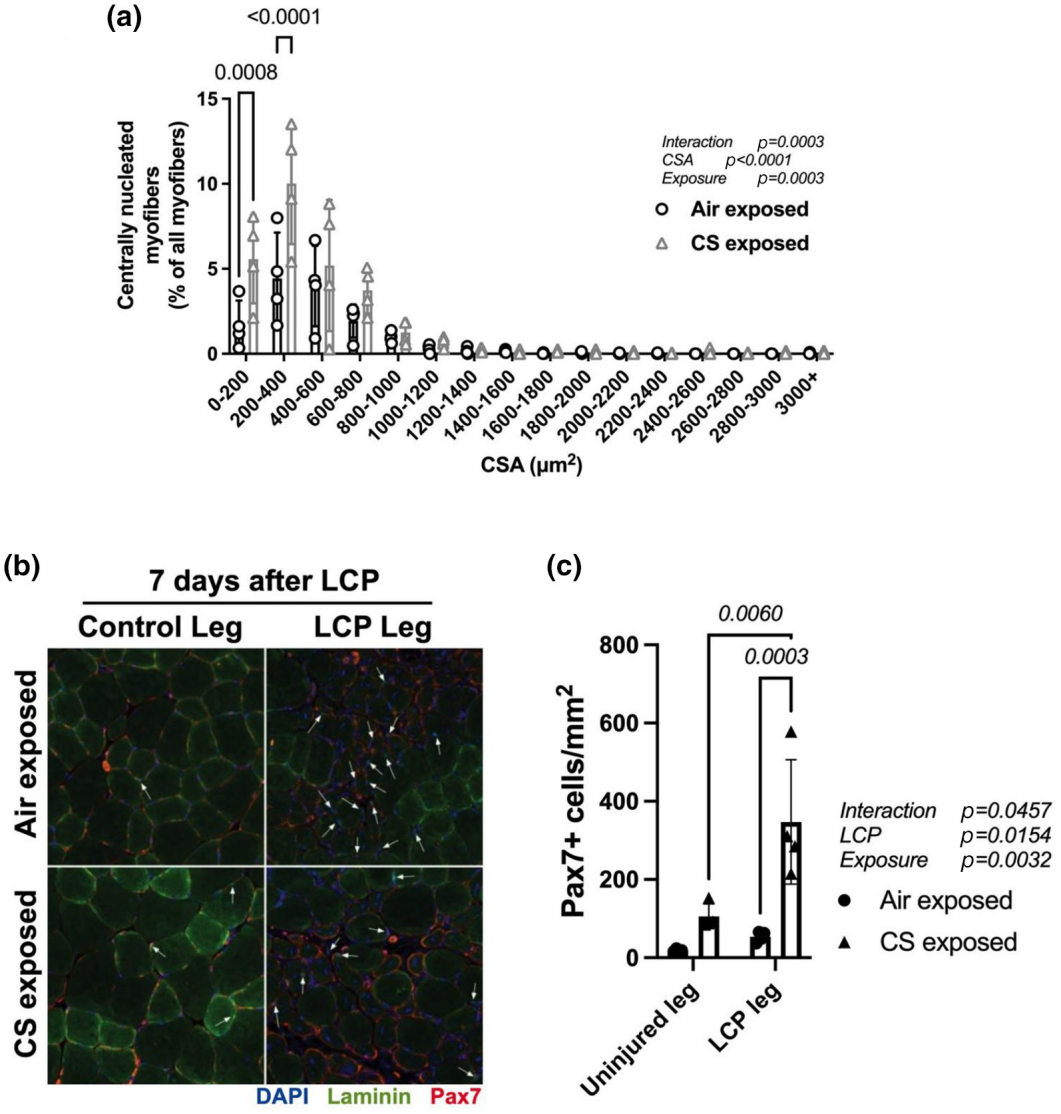
Detection of centrally nucleated myofibers and Pax7+ cells from TA muscle sections 7 days after lengthening contractions in Air‐ and CS‐exposed mice. (a) Comparison of centrally nucleated myofiber CSA distribution between Air and CS exposure groups in muscle sections from LCP legs; (b) Representative immunofluorescence image of TA muscle sections immunostained with laminin (red), nuclei (DAPI) and Pax7 (green) from control and LCP legs from Air and CS exposed groups. Scale bar = 100 μm; (c) Number of Pax7+ cells relative to the area (in mm^2^) of each muscle section. *N* = 4 mice per group, two‐way ANOVA, Air versus CS groups. *p‐*values obtained by the Bonferroni post‐test are shown above bars.

Compared to the total number of myofibers, there was a higher number of centrally nucleated myofibers with CSA of <400 μm^2^ in the CS exposed group compared to the Air exposed group (*p* = 0.0008 and *p* < 0.0001, for 0–200 and 0–400 μm^2^, respectively, Bonferroni post‐test, Figure 3a). As represented in Figure 3b, muscle sections from LCP‐subjected hindlimbs from both exposure groups show a greater number of Pax7+ cells compared to muscle sections from non‐injured (control) legs. When the number of Pax7+ cells in muscle sections from CS‐exposed are compared to Air‐exposed groups, there is an exposure effect (*p* = 0.0032, two‐way ANOVA) with CS‐exposed mice presenting a greater number of Pax7+ cells (106 ± 32 cells/mm^2^) than air‐exposed mice (19 ± 3 cells/mm^2^) in uninjured muscles. In muscles subjected to LCP, the increase in Pax7+ cells in CS‐exposed mice was significantly different than air‐exposed mice (*p* = 0.0003, Bonferroni post‐test), and there was an interaction effect between exposure and LCP (*p* = 0.0457, Two‐way ANOVA) (Figure 3c).

### 3.4 Cytokines and myogenic factor mRNA levels in uninjured and regenerating EDL

**FIGURE 4:**
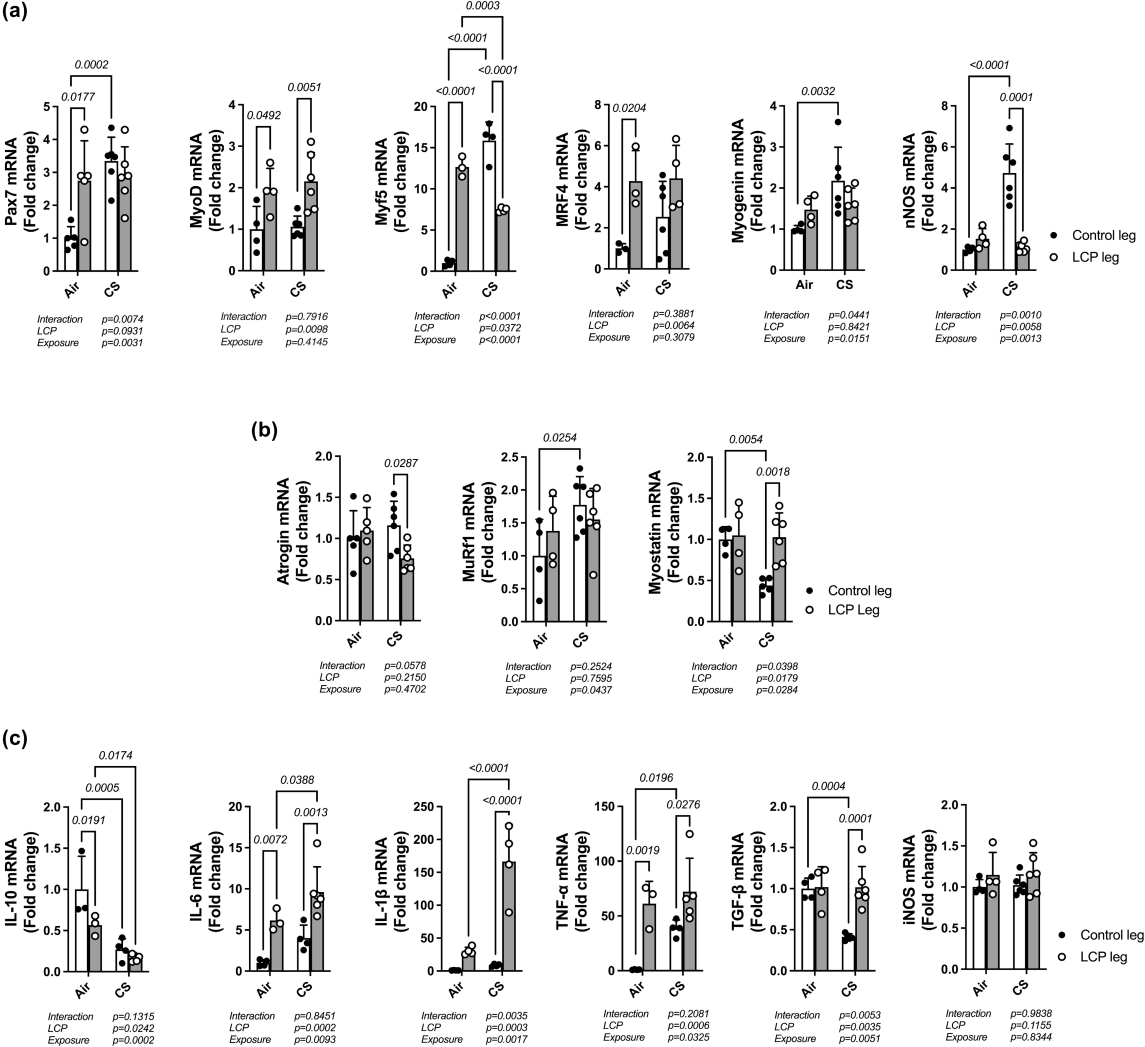
Changes in mRNA expression of myofiber repair and maturation (a), muscle wasting (b), and inflammatory (c) genes in EDL muscles from control and LCP legs from both exposure groups. Data was normalized by the mRNA expression measured in control muscles from Air‐exposed mice. *N* = 3–6 mice per sample, two‐way ANOVA. *p*‐values obtained by the Bonferroni post‐test are shown above bars.

Levels of mRNA for genes associated with myofiber repair and maturation (Figure 4a), muscle wasting (Figure 4b), and inflammation (Figure 4c) were measured in the EDL by RT‐PCR. LCP injury increased mRNA levels of *Pax7* (2.7‐fold, *p* = 0.009), *MyoD* (1.9‐fold, *p* = 0.049), *Myf5* (13‐fold, *p* < 0.0001), and *MRF4* (4.2‐fold, *p* = 0.04) in the Air group (LCP vs. control, Bonferroni post‐test, Figure 4a). *Pax7* (3.3‐fold, *p* = 0.0005), *Myf5* (16‐fold, *p* < 0.0001), *myogenin* (2‐fold, *p* = 0.006) and *nNOS* (4.7‐fold, *p* < 0.0001) mRNAs were increased in the uninjured CS groups in (Air vs. CS, Bonferroni post‐test). Interestingly, in muscles from CS‐exposed mice, *nNOS* (4.7‐fold, *p* < 0.0001) and *Myf5* (2.3‐fold, *p* < 0.0001) mRNAs were decreased by LCP compared to uninjured muscles (Control vs. LCP, Bonferroni post‐test).

In measurements of gene expression associated with muscle wasting, CS exposure increased *MuRf1* (1.8‐fold, *p* = 0.050) and decreased *myostatin* (2‐fold, *p* = 0.011) mRNA levels compared to the Air group (Air vs. CS, Bonferroni post‐test, Figure 4b). Injury by LCP prevented these effects (Figure 4b).

For genes related to muscle inflammation, *IL‐6* (6‐fold, *p* = 0.01), *IL‐1β* (30‐fold, *p* = 0.002), and *TNF‐α* (60‐fold, *p* = 0.004) mRNA levels increased, and *IL‐10* (2‐fold, *p* = 0.04) decreased, in EDL muscles subjected to LCP in Air exposed mice (Control vs. LCP, Bonferroni post‐test, Figure 4c). In CS exposed mice, non‐injured muscle *TNF‐α* mRNA levels increased by 39‐fold (*p* = 0.04), while *IL‐10* and *TGF‐β* decreased by 3.7‐fold (*p* = 0.0009) and 2.5‐fold (*p* = 0.0008), respectively (Air vs. CS, Bonferroni post‐test, Figure 4c). Only *IL‐1β* mRNA levels were increased (5.5‐fold, *p* = 0.0004) by LCP injury in CS exposed mice (Air vs. CS, Bonferroni post‐test, Figure 4c). The mRNA levels of *iNOS* were not different between injury or exposure groups.

### 3.5 Recovery of EDL mass and force

EDL muscles from both hind limbs were isolated after 7 days of recovery from LCP. EDL mass was not different between non‐injured and LCP from Air‐exposed mice (13.6 ± 1.3 mg vs. 12.5 ± 2.0 mg, respectively, *p* = 0.15, Bonferroni post‐test). However, in CS‐exposed mice, EDL mass was lower in the LCP‐injured muscles (11.8 ± 2.0 mg) than the contralateral, non‐injured EDL (13.3 ± 1.2 mg, *p* = 0.04, Bonferroni post‐test).

**FIGURE 5:**
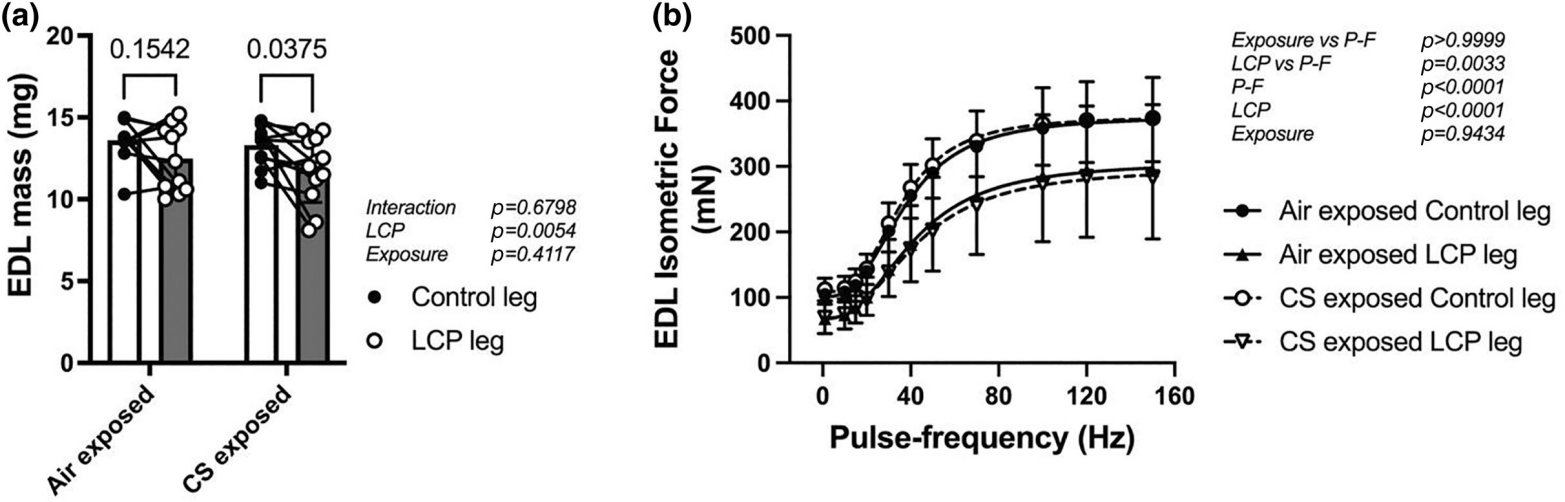
Extensor digitorum longus (EDL) muscle morphometrics and force production 7 days after lengthening contractions in Air and CS exposed mice. (a) EDL mass from legs subjected to LCP (LCP leg) and from contralateral non‐injured legs (control leg). *N* = 11 mice for Air exposed and 12 mice for CS exposed groups, two‐way ANOVA repeated measures. *p‐*values obtained by the Bonferroni post‐test are shown above bars. (b) Force production evoked by different frequencies of pulse‐stimulation in muscles from control and LCP legs. *N* = 11 mice for Air exposed and 12 mice for CS exposed groups, three‐way ANOVA repeated measures.

Force‐frequency curves collected from LCP‐injured EDL from both Air‐ and CS‐exposed groups, showed lower absolute forces at all frequencies of pulse‐stimulation than the contralateral, non‐injured EDL (Air‐exposed, −27 ± 22%, CS‐exposed, −30 ± 21%; *p* < 0.0001, Control vs. LCP, three‐way ANOVA, Figure 5b). However, maximal force was not different between Air and CS groups for both non‐injured and LCP‐injured EDL (*p* = 0.943, Air vs. CS, three‐way ANOVA).

### 3.6 In vivo measurements of lung compliance

**FIGURE 6:**
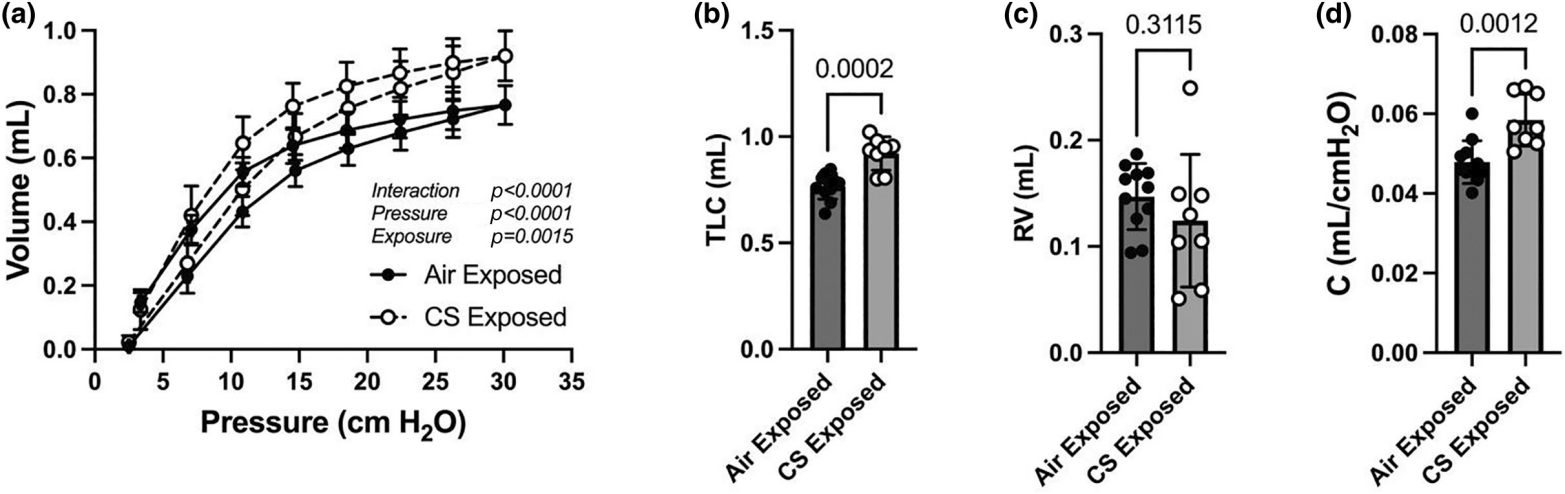
Lung mechanical properties of mice exposed to CS or Air for 4 months. (a) In vivo lung pressure‐volume responses. Two‐way ANOVA; (b) total lung capacity (TLC); (c) residual volume (RV); (d) lung compliance (C). Student's *t*‐test *p*‐values are shown above bars in b–d. *N* = 11 mice for Air exposed and 8 mice for CS exposed groups.

Static lung mechanics were evaluated at the end of the 4‐month CS‐ (or Air‐) exposure period. As shown in Figure 6a, there was a clear increase in lung volumes in the inflation and deflation phases in CS‐exposed mice at pressures above 10 cmH_2_O (*p* = 0.0015; Two‐way ANOVA, *p < 0.01 vs. Air exposed, Bonferroni post‐test, Figure 5c). At 30 cmH_2_O, CS‐exposed mice had ~20% greater the TLC than Air‐exposed mice (*p* = 0.0002, Student *t*‐test, Figure 6b). RV were not different between groups (*p* = 0.31, Student *t*‐test, Figure 6c). Compliance was ~20% higher in the CS group vs. Air group (*p* = 0.001, Student *t*‐test, Figure 6d).

## 4 DISCUSSION

The present investigation is the first to implement an eccentric exercise‐induced muscle injury using nerve‐stimulated lengthening contractions to examine the effects of CS exposure on muscle regeneration. In this study, we found that long‐term (4 months), daily exposures of male mice to CS: (1) prevented a full recovery of mass and myofiber cross‐sectional area after a single set of lengthening contractions in fast‐twitch locomotor muscles; (2) resulted in smaller myofibers with central nuclei and a greater number of Pax7+ cells, indicating inefficient repair; and (3) signaled satellite cell pre‐activation even before lengthening injury. These data suggest that CS exposure diminishes the response to recovery after exercise‐induced injury.

The mechanisms that explain the decrement of muscle mass and force in smokers (Batty & Zaninotto, 2018; Carrasco‐Rios et al., 2019) and COPD patients (Seymour et al., 2010) have been extensively examined. Conversely, it has only recently been appreciated that muscle regenerative capacity may also be impaired. A study performed in MuSC isolated from *vastus lateralis* of COPD patients showed slower proliferation and differentiation compared to healthy subjects (Theriault et al., 2014). These findings suggest a poor regenerative capacity of muscle progenitor cells in COPD. Most recently, the effects of smoking on muscle recovery from injury were investigated in mice in which muscles were damaged with BaCl_2_, a procedure that causes massive myofiber degeneration (Chan et al., 2020). The authors found that chronic CS exposure slowed the recovery muscle force and reduced MuSC number, associated with an exaggerated proinflammatory response during the early phase of muscle regeneration (Chan et al., 2020). However, until this present investigation, it was not known whether chronic tobacco smoke exposure, eccentric exercise, or a combination of these conditions could affect muscle recovery from eccentric‐exercise muscle injury.

The present work investigated the effects of smoke exposure on the early phase of muscle recovery by subjecting locomotor hindlimb muscles of experimental mice to nerve‐stimulated lengthening contractions. Unlike BaCl_2_‐induced injury, in which decrements in force are completely due to myofiber degeneration, only 50% of force decay after lengthening contractions is attributed to myofiber degeneration (Rathbone et al., 2003). Therefore, nerve‐stimulated lengthening contractions model the injury experienced during strenuous or eccentric activity. In the present study, lengthening contractions produced approximately a 70% reduction in maximal torque at the end of the contraction bout, and this decrease in torque was similar for both exposure groups. The LCP‐produced decrement in torque development has been attributed to multiple causes. These include alterations in intramyofiber force‐bearing structures, failure in excitation‐contraction coupling, physical disruption of myofiber membranes that lead to myofiber degeneration (Rathbone et al., 2003; Warren et al., 2001), and, importantly, muscle fatigue (Morgan et al., 2004). Although we have not determined whether muscle injury produced by the LCP was different between both groups, the similar decrements in torque in both exposure groups during LCP suggest that both groups of mice received the same damaging stimuli.

It has been shown that near‐complete muscle recovery from lengthening contractions typically occurs after 28–35 days post‐injury (Rathbone et al., 2003; Warren et al., 2001). Thus, the seventh day is considered an early phase of recovery from lengthening contractions, and a sign of muscle repair is the presence of centrally nucleated myofibers. Central nuclei occur when new myofibers are generated (Tierney et al., 2018). Also, they are present when focal injury does not lead to myofiber necrosis (Roman et al., 2021). However, new or regenerating myofibers have much smaller cross‐sectional areas than mature myofibers (Schroer et al., 2019). Our data show that a combination of CS and LCP injury reduces the cross‐sectional area of centrally nucleated myofiber on the seventh day of muscle recovery. At the same time, CS exposure alone did not have an effect (Figure 2). This was supported by the specific reduction in EDL muscle mass in LCP‐injured limb from CS‐exposed mice (Figure 5). This suggests that the process of growing regenerating myofibers is impaired or there are a greater number of newly formed myofibers during regeneration. This is not surprising since it has been previously demonstrated that muscle biopsies obtained from COPD patients with relatively preserved muscle mass had about twice as many centrally nucleated myofibers (Theriault et al., 2012) than non‐COPD muscle. Several months of daily CS exposure have been reported to lead to several changes in locomotor muscles. The CS‐exposure muscle phenotype includes greater pro‐inflammatory signaling (Tang et al., 2010), muscle oxidative stress (Barreiro et al., 2012), lower capillarity (Gosker et al., 2009), myofiber contractile dysfunction (Nogueira et al., 2018; Rinaldi et al., 2012), and neuromuscular junction denervation (Kapchinsky et al., 2018). Thus, these changes in muscle structure and function from CS exposure would be expected to persist during and influence the regeneration phase.

When muscle injury leads to myofiber degeneration, a well‐orchestrated and highly regulated inflammatory response involving multiple cell types that leads to the activation, proliferation, and differentiation of MuSC to form new myofibers (Tidball, 2011). We found that TA muscle from uninjured legs (CS exposure alone) showed a greater number of Pax7+ cells compared to air‐exposed mice. The number was higher when CS and LCP injury were combined (Figure 3c). Also, *Pax7* gene expression, which controls MuSC proliferation, was 3× increased by CS exposure alone and was not affected by a combination of CS and LCP injury (Figure 4). These data suggest that CS exposure increases satellite cell proliferation in muscles not subjected to lengthening injury but does not impair the injury‐dependent activation of satellite cell proliferation. Analysis of *Myf5* gene expression, known to control MuSC quiescence, was also significantly increased (15×) by CS exposure alone, and there was a suppression in expression by LCP in CS‐exposed mice. Also, there was no difference in MyoD expression in uninjured muscles between exposure groups. Both *Pax7* and *Myf5* remain highly expressed during MuSC activation after muscle injury (Kuang et al., 2007; Zammit, 2017). Although in the present work, mice were exposed to CS for 4 months, our group has shown before that mice exposed to CS for 2 months have a lower number of Nestin+‐Pax7+ (i.e., quiescent) cells around myofibers (Nogueira et al., 2018), which could result from a lower MuSC self‐renewal. Thus, the increase in Pax7+ cells, together with the upregulation of *Pax7* and *Myf5* expression in uninjured muscles detected in the present study from CS‐exposed mice, suggest that CS exposure leads to a greater number of committed myoblasts in the uninjured leg. This is further increased with lengthening injury. This may interfere with the initial process of MuSC activation and proliferation during muscle repair. However, we have not tested whether smoke exposure alters MuSC function, and further investigation is needed.

CS exposure alone also upregulated *myogenin* mRNA levels. Although this gene is normally increased in early‐phase recovery from eccentric contractions and not in uninjured muscles (Lovering et al., 2007), the effects of chronic CS exposure are unknown. *Myogenin* gene is expressed in differentiated MuSC (i.e., myoblasts) and regulates myotube formation (Ganassi et al., 2020). Myogenin has been reported to be upregulated in uninjured muscle deficient in hypoxia‐induced factor 2A, and this was accompanied by a premature myogenic differentiation of MuSC (Xie et al., 2018). Furthermore, mRNA level of *nNOS*, which is a marker of myofiber maturation and growth (Anderson, 2000; Montagna et al., 2019), was also upregulated by CS alone and decreased by a combination of CS and LCP injury. Pharmacological inhibition of nNOS (Anderson, 2000) or *nNOS* gene ablation (Church et al., 2011) in mice after crush‐ or myotoxin‐injury, respectively, leads to an incomplete myofiber regeneration and a smaller muscle cross‐sectional area during the repair process. Therefore, the suppression of *myogenin* and *nNOS* expression by LCP in CS‐exposed mice indicates that smoke exposure may also impair myofiber maturation and growth after lengthening contractions and suggest that CS exposure induces myogenic differentiation independent of lengthening injury. Alternatively, the increase in myogenic gene expression in uninjured muscles of CS‐exposed mice could be due to chronic insults produced by toxic components present in CS. CS components have been shown to directly impair muscle contractile function, reduce the number of myofiber‐associated satellite cells (Nogueira et al., 2018), and cause muscle atrophy (Zhang et al., 2013).

After muscle injury, several cytokines (e.g., TNF‐α (Chen et al., 2007)) are transiently released by local inflammatory cells (i.e., neutrophils and macrophages) and fibroadipogenic progenitor (FAP) cells at the injury site, which signal MuSC proliferation (Dumont et al., 2015; Lemos et al., 2015; Tatsumi et al., 2009). We found that mRNA levels of several pro‐inflammatory cytokines (*IL‐6, IL‐1β*, and *TNF‐α*) were either increased by CS alone or a combination of CS and LCP injury. Our group has previously demonstrated that life‐long lung‐specific *TNF‐α gene* overexpression in mice (SP‐C/TNF‐α), a mouse model with persistent pulmonary inflammation (Tang et al., 2013; Zuo et al., 2011), leads to locomotor muscle weakness and reduced muscle mass. In CS‐exposed mice, lung and locomotor muscles have sustained expression of pro‐inflammatory cytokines (Tang et al., 2010), and excessive TNF‐α signaling in muscle reduces myofilament response to Ca^2+^ transients in intact single myofibers (Reid et al., 2002). Furthermore, intact single myofibers obtained from mice treated with CS extract for 2 months show slowed Ca^2+^ uptake by the sarcoplasmic reticulum, which is exaggerated during repetitive contractions (Nogueira et al., 2018). Although there were no differences in the EDL force recovery during the early‐stage recovery from lengthening contractions injury between the two exposure groups, it is not known whether intracellular Ca^2+^ handling and myofilament function would be changed at later stages of the recovery period. In addition, Langen et al. (Langen et al., 2006) showed impaired recovery of muscle mass and myosin heavy chain content following unloading‐reloading muscle injury in both gastrocnemius and soleus muscles of SP‐C/TNF‐α mice. Excessive pro‐inflammatory conditions are known to impair the function of MuSC and FAP cells (Talbert & Guttridge, 2016). This suggests that an excessive pro‐inflammatory response produced by CS exposure may interfere with the interplay between myogenic progenitor cells and local inflammatory cells during regeneration. Taken together, these data suggest that smoke exposure delays the restoration of muscle and myofiber size after eccentric exercise‐induced muscle injury by over‐activating pro‐inflammatory cytokines that derail the repair machinery.

## 5 CONCLUSION

In summary, we found that daily CS exposure periods over the course of 4 months in male mice impair early‐stage myofiber growth recovery after lengthening contractions. This may be due to an excessive pro‐inflammatory response in regenerating muscle that decreases muscle repair responsiveness, thereby slowing early‐stage muscle recovery from injury. These data support the prolonged recovery after strenuous exercise in tobacco smokers and COPD patients.

### 5.1 Limitations of the study

In the present study, the muscle morphometric and isolated force analyses were obtained from different muscles (i.e., TA and EDL, respectively). Ex‐vivo functional analysis in isolated muscles requires the use of muscles with smaller cross‐sectional areas than what is found in TA muscles to avoid diffusion limitation of oxygen and, therefore, anoxic cores, during contractions (Barclay, 2005). However, the contribution of both TA and EDL muscles to torque development in response to peroneal nerve stimulation is strikingly different (i.e., 89% and 11%, respectively) (Warren et al., 1999). Future experiments that investigate the consequences of smoke exposure on force recovery of the TA muscles will require in vivo measurements of muscle torque over the recovery period.

Furthermore, the present investigation focused on the effects of CS exposure on the early phase of recovery from lengthening contractions, which recovery from exercise‐induced injury is far from complete. Satellite cells obtained from COPD patients have a slower differentiation rate (Theriault et al., 2014), and differences in force recovery would possibly be detected only at laters stages of recovery. Therefore, it is also possible that smoking interferes with muscle regeneration in the second half of the myofiber recovery process, which will require further inquiry.

## AUTHOR CONTRIBUTIONS

N. E. S., M. L., F. C. G. R., E. C. B., A. S. and L. N. contributed to the conception, design, and interpretation of the data. N. E. S., M. L., I. R.‐S., F. C. G. R., and L. N. performed the experimental work and analyzed the data. E. C. B. and L. N. wrote the manuscript. All authors approved the final version of the manuscript.

## FUNDING INFORMATION

This research was supported by the Tobacco‐Related Disease Research Program (TRDRP) New Investigator Award T29KT0397 and TRDRP Research Award T32IR5221 (to L.N.). The UCSD Light Microscopy Facility is supported by the National Institute of Neurological Disorders and Stroke Grant NS047101.

## CONFLICT OF INTEREST STATEMENT

No conflicts of interest, financial or otherwise, are declared by the author(s).

## Data Availability

The data that support the findings of this study are available from the corresponding author upon reasonable request.
